# Mulberry (*Morus alba* L.) leaf polysaccharide ameliorates insulin resistance‐ and adipose deposition‐associated gut microbiota and lipid metabolites in high‐fat diet‐induced obese mice

**DOI:** 10.1002/fsn3.2689

**Published:** 2021-12-24

**Authors:** Xin Zhao, Zhifei Fu, Minghe Yao, Yu Cao, Tongtong Zhu, Rui Mao, Ming Huang, Yafen Pang, Xianghui Meng, Lin Li, Boli Zhang, Yuhong Li, Han Zhang

**Affiliations:** ^1^ State Key Laboratory of Component‐based Chinese Medicine Tianjin University of Traditional Chinese Medicine Tianjin China; ^2^ Key Laboratory of Pharmacology of Traditional Chinese Medical Formula Ministry of Education Tianjin University of Traditional Chinese Medicine Tianjin China

**Keywords:** gut microbiota, insulin resistance, lipids, metabolic disorder, mulberry leaf polysaccharide, obesity

## Abstract

Dietary supplements are currently being used to ameliorate metabolic alterations via maintaining gut microflora balance. Mulberry leaf is known as medicine homologous food for its glucose‐ and lipid‐modulating properties. However, the effects of mulberry leaf polysaccharide (MP) on metabolic dysbiosis and gut microbiota are still poorly understood. After extraction and characterization, the beneficial effects of water‐soluble MP were evaluated in high‐fat diet‐induced obese mice. MP treatment could reduce adipose tissue, improve insulin resistance, and alleviate the pathological lesions in colon. Investigation of the underlying mechanism showed that MP modulated gut microbial community by 16S rRNA analysis and reversed the elevation of lipid indexes by plasma lipidomics analysis. Correlation analysis indicated that the abundance of seven key bacterial species and six lipids were closely associated with the metabolic traits, respectively. Overall, MP could ameliorate metabolic disorders, and modify the gut microbiota and lipids. This would greatly facilitate the utilization of MP as a functional food.

## Graphical abstract

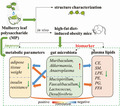

## 1 INTRODUCTION

The leaf of mulberry (*Morus alba* L.), belonging to the family *Moraceae*, is one of the most commonly used traditional Chinese medicines (TCMs) and also functional foods to treat diabetes and its complications (Lown et al., 2017; Riche et al., 2017). Phytochemical and pharmacological studies revealed that the therapeutic effect of mulberry leaf is owed to a variety of active compounds, such as polysaccharides, flavonoids, polyphenols, and alkaloids (Ge et al., 2018; Li et al., 2019; Meng et al., 2020; Wang et al., 2021; Zhao et al., 2019). Mulberry leaf tea has recently received much attention as a dietary supplement with remedial effects (He et al., 2018). The water‐soluble polysaccharide from mulberry leaf (MP), one of the main bioactive compounds, has been proved to exhibit excellent hypoglycemic, antiinflammatory, antioxidant, immunomodulating, modulating gut microbiota, and antirenal fibrosis properties (Chen et al., 2021; Khan et al., 2019; Wang et al., 2020; Wu, 2019; Zhang et al., 2019). Especially, MP improves glucose metabolism and insulin resistance (IR) in type 2 diabetic rats (Ren et al., 2015). However, the pharmacological mechanism of this activity remains to be elucidated.

The microbiota‐mediated dietary interventions can also be used for the prevention and treatment of metabolic syndrome such as IR, obesity, and diabetes (Chambers et al., 2019). The pilot study by Del Chierico et al. (2021) presented a reduced bacterial richness of gut microbiota in patients with IR. In individuals with metabolic complications, there is a decreased abundance of *Bifidobacterium* and *Akkermansia*, and an increase in Firmicutes/Bacteroidetes (F/B) ratio (Vallianou et al., 2019). Many polysaccharides have been reported to improve parameters of the metabolic disorders in animal models through alterations in the gut microbiome by decreasing the ratio of F/B (Chen et al., 2020; Gu et al., 2020; Shi et al., 2015). In vitro bacteria–drug interaction indicated that both human *Bacteroides cellulosilyticus* and *Bacteroides ovatus* could generate acetate and propionate by utilizing MP (Wang et al., 2020). Zhao et al. (2019) reported that MP decreased gut *Escherichia coli* and increased gut lactobacilli and bifidobacterial in early weanling pigs. The regulation of MP on the general bacterial composition at the phylum levels (i.e., F/B) and genus levels remains an open question. Therefore, we tried to study the potential mechanism of MP in alleviating IR and metabolic disorders from the perspectives of gut microbiota.

Excessive dietary fat intake aggravates the development of IR, obesity, and type 2 diabetes, which are often accompanied by lipid disorders. The levels of lysophospholipids (LPs), phosphatidylcholine plasmalogens (PC‐PLs), sphingomyelins (SMs), and cholesterol esters (CEs) were associated with lower diabetes risk, while the profile of triacylglycerols (TAGs), diacylglycerols (DAGs), and phosphatidylethanolamines (PEs) was associated with higher diabetes risk (Razquin et al., 2018). Mass spectrometry‐based approaches in lipidomics analysis provide a rapid, comprehensive method for the determination of lipid metabolism and could provide predictive capabilities for biomarker related to diseases, rather than total cholesterol (TC) and triglyceride (TG) routine test (Hu & Zhang, 2018). The discovery of characteristic lipids of MP supplement by lipidomics technology is feasible and worthwhile, and the subsequent identification and isolation of lipid molecules can attract pharmacological interest. Furthermore, gut microbiota might be also likely to influence lipid metabolism (Matey‐Hernandez et al., 2018).

In our study, the beneficial effects and potential mechanisms of MP were evaluated in HFD‐fed mice by detecting metabolic parameters, gut microbiota composition, and plasma lipidomics. Moreover, we revealed the relationships among the abundance of gut microbiota, lipidomics, and metabolic changes induced by MP. With the supports of the obtained results, we propose that MP could be developed as an essential dietary supplement to treat IR, improve gut microbiota, and lipid metabolism.

## 2 MATERIALS AND METHODS

### 2.1 Materials and reagents

Mulberry leaf was purchased from Tong Ren Tang Co. (Beijing, China, Series: 20180605). Standards of monosaccharide include d‐Mannose (Man), l‐Rhamnose (Rha), d‐Glucuronic acid (GlcA), d‐Galacturonic acid (GalA), d‐Glucose (Glc), d‐Galactose (Gal), d‐Xylose (Xyl), and l‐Fucose (Fuc) were purchased from Sigma‐Aldrich. Phenyl‐3‐methyl‐5‐pyrazolone (PMP), trifluoroacetic acid (TFA), sodium hydroxide (NaOH), and monopotassium phosphate (KH_2_PO_4_) were purchased from Aladdin. The purity of each standard was no <98%. Chromatographic grade acetonitrile and formic acid were purchased from Fisher Scientific. Potassium phosphate buffer (PBS) was purchased from Solarbio. The other chemical reagents were of analytical grade and purchased from Sinopharm Chemical Reagent Co., Ltd.

### 2.2 Preparation mulberry leaf polysaccharides

In short, 1.0 kg of mulberry leaf was dried and smashed to powder, then soaked overnight with 95% ethanol to remove some chlorophyll. The residue was dried and extracted with 12 L water for 2.0 h at 100°C. The supernatant was centrifuged at 3500 g for 10 min, and the residue was extracted again with 10 L water for 2.0 h. The extract was concentrated to one‐fifth of volume by vacuum rotary evaporation, then 95% ethanol was added until the final concentration of ethanol was 80%, standing 24 h at 4°C. Then, the precipitation was collected and freeze dried to obtain MP. The primary composition of MP was characterized by detection of total sugar and protein content (Bitter & Muir, 1962; Bradford, 1976), molecular weight (Sun et al., 2009), and monosaccharide component (Dai et al., 2010).

### 2.3 Animals

Six‐week‐old male C57BL/6N mice were purchased from Beijing Vital River Laboratory Animal Technology Co., Ltd, and housed in a controlled condition (22 ± 2°C, 40%–60% humidity, and 12 h light–dark cycle). After 1 week of adaptation, the mice were randomly divided into control group (Con), HFD group, and MP group (*n* = 8–10). The mice in the Con group were fed with standard diet (13.5% of energy from fat; 1022; Beijing HFK Bioscience Co. Ltd) and other mice were fed with a HFD (60% of energy from fat; H10060; Beijing HFK Bioscience Co. Ltd). Mice in the MP groups were, respectively, gavaged with high dose of MP solution (HMP‐100 mg kg^−1^ day^−1^) and low dose of MP solution (LMP‐50 mg kg^−1^ day^−1^), and other mice were administrated with water as control. After 20 weeks, the animals were subjected to the metabolic cages, and food intake data were collected over 24 h. Feces were collected for 16S sequencing analysis. The animals were fasted for 10 h prior to euthanasia, and blood samples and vital organs were collected. This study was approved by the Laboratory Animal Ethics Committee of Tianjin University of Traditional Chinese Medicine (TCM‐LAEC2020049).

### 2.4 Metabolic evaluation

#### 2.4.1 Body weight and organs weight measurements and histological examination

Body weight was recorded weekly during this experiment. Twenty‐four‐hour calorimetry intake data were calculated. Freshly isolated brown adipose, perirenal adipose, subcutaneous adipose, epididymal adipose, and liver were weighed after the sacrifice of mice. Paraffin wax‐embedded liver tissue and epididymal adipose were stained using hematoxylin and eosin (H&E) or Oil Red O as described previously (Xu et al., 2019).

#### 2.4.2 Fasting blood glucose (FBG), fasting insulin (FIN), leptin, and glucose tolerance test (GTT)

Mice were fasted overnight with free access to water, FBG was determined by ACCU‐CHEK^®^ Performa Glucometer and compatible blood glucose test strips. GTT was performed after glucose administration intraperitoneally (2.0 g kg^−1^ of body weight), blood glucose concentrations were measured from tail vein blood at 0, 15, 30, 60, 90, and 120 min, and the area under the curve (AUC) of GTT (GTT AUC) was calculated. Fasting insulin (FIN) and leptin levels were measured using MILLIPLEX^®^ MAP kits (Merck Milliplex). The index of insulin resistance was calculated by the following formulas: homeostasis model assessment of insulin resistance (HOMA‐IR) = FIN × FBG (mM)/22.5.

#### 2.4.3 Other biochemical measurements

Plasma total cholesterol (TC) and triglyceride (TG) were measured by Automatic Biochemistry Analyzer (Hitachi 7020) according to the manufacturer's instructions. Free fatty acid (FFA) level in plasma was detected by chemical colorimetric. Levels of plasma lipopolysaccharide (LPS) were analyzed via enzyme‐linked immunosorbent assay (ELISA) kits from Linco Research (Shanghai Huyu Biotechnology Co., Ltd).

### 2.5 Colonic permeability detection

After the mice were sacrificed, the colonic segments were cut out and fixed with phosphate‐buffered formalin and embedded in paraffin. Then, the sections were stained with H&E (Song et al., 2018). The change in zonula occludens‐1 (ZO‐1) protein in colonic tissue was determined with immunofluorescence staining. Briefly, paraffin‐embedded sections of colon tissue were prepared and incubated with antibodies according to Xie et al. (2021). Sections were counterstained with DAPI and observed using fluorescence microscopy (Nikon). Fluorescence images were merged and analyzed by Image J.

### 2.6 Microbial diversity analysis

#### 2.6.1 Fecal sample collection, DNA extraction, and sequencing

Fresh fecal samples (200 mg of each) were collected under sterile condition and immediately frozen at −80°C before euthanasia. Afterward, the genomic DNA was extracted by CTAB/SDS method and the concentration of each DNA sample was quantified to 1 ng μl^−1^. The library of 16S rRNA gene V4 region (primer: 515F‐806R) was constructed, qualified, and sequenced (Novogene) on an Illumina HiSeq 250 platform following the procedure in Fu et al. (2020).

#### 2.6.2 Sequencing analysis of the gut microbiota composition

Raw reads from the original DNA fragments were quality filtered and merged by FLASH (Magoč & Salzberg, 2011). Sequences were analyzed using Qiime software package (Caporaso et al., 2010). Operational taxonomic units (OTUs) were picked by aligning sequences with ≥97% similarity cutoff using *de_novo_otus.py*, and then annotated by SILVA database. Alpha diversity index chao1 and abundance‐based coverage estimator (ACE) estimate the species abundance; and observed species estimates the amount of unique OTUs found in each sample, and Shannon index was generated based on these three metrics using Qiime (Version 1.9.1). Weighted UniFrac for principal coordinate analysis (PCoA) was computed for beta diversity analysis using R packages ggcorrplot (Version 2.15.3). Correlations between the key microbial phylotypes and metabolic‐related parameters were calculated by Spearman correlation analysis using R packages psych (Version 2.15.3).

### 2.7 Lipidomics analysis

#### 2.7.1 Plasma samples preparation

Plasma samples were prepared using Sarafian method with some modifications (Sarafian et al., 2014). Briefly, 100 μL plasma sample was placed on ice, and then extracted by addition of 300 μL cold isopropanol and vortexed for 5 min. The emulsion was allowed to stand at −20°C for 1 h and centrifuged at 13,200 g for 10 min. The upper isopropanol layer phase was collected in a fresh Eppendorf tube, and 2 μl was injected for ultrahigh‐performance liquid chromatography‐mass spectrum (UHPLC‐MS) analysis. To evaluate the repeatability and robustness of the instrumental system, quality control (QC) was performed. Ten milliliter injection sample was pooled as QC sample together with validated standards.

#### 2.7.2 Instrument conditions and data analysis

The separation was performed by UHPLC. An AB SCIEX QTRAP mass spectrometer (6500+, AB Sciex) was applied for data acquisition. The detailed conditions of UHPLC‐QTRAP‐MS/MS were presented as described previously (Tan et al., 2021).

The SCIEX OS software was used for the raw data processing. Principal component analysis (PCA), partial least squares‐discrimination analysis (PLS‐DA), and orthogonal PLS‐DA (OPLS‐DA) were performed by SIMCA‐P software (version 14.1; Umetrics AB). Par was used for data normalization before multivariate analysis. Potential biomarkers were selected according to the variable importance in the projection (VIP > 1) from the PLS‐DA model and Student's *t*‐test (*p* < .05).

### 2.8 Statistical analysis

Data were expressed as mean ± SEM. All statistical analyses were performed by SPSS 17.0 software. One‐way ANOVA with Tukey's post hoc test or a Kruskal–Wallis test followed by Dunn's multiple comparison was used for comparisons among multiple groups. Student's *t*‐test was used for comparisons of two groups. Differences with *p* < .05 were considered statistically significant. GraphPad Prism 5.0 (GraphPad Software Inc.) was used for the graphical work.

## 3 RESULTS

### 3.1 Preliminary characterization of MP

The results of phenol–sulfuric acid assays showed that MP contained 35.72% ± 0.035% carbohydrates. The content of uronic acid of MP determined via carbazole sulfate method was 2.62% ± 1.19%. The protein content of MP was 12.81% ± 0.66%. This result showed that MP was acidic polysaccharide. The molecular weight distribution of MP was analyzed using high‐performance gel permeation chromatography (HP‐GPC) technique and the result was shown in Figure S1a. Molecular weight chromatography of MP showed two main peaks, including one peak covering from 10.9 min to 12.5 min with molecular weight of 203–838 kDa; the other one covering a wide range peak from 12.5 to 19.8 min with molecular weight of 0.3–203 kDa. In addition, MP was composed of eight monosaccharides, including mannose, rhamnose, glucuronic acid, galacturonic acid, glucose, galactose, arabinose, and fucose, at a molar ratio of 1.00:1.16:0.80:2.11:5.12:2.37:2.16:0.73 (Figure S1b).

### 3.2 MP exhibits metabolic improved effects in HFD‐fed mice

#### 3.2.1 MP alleviates body, liver, and adipose tissue weight

**FIGURE 1:**
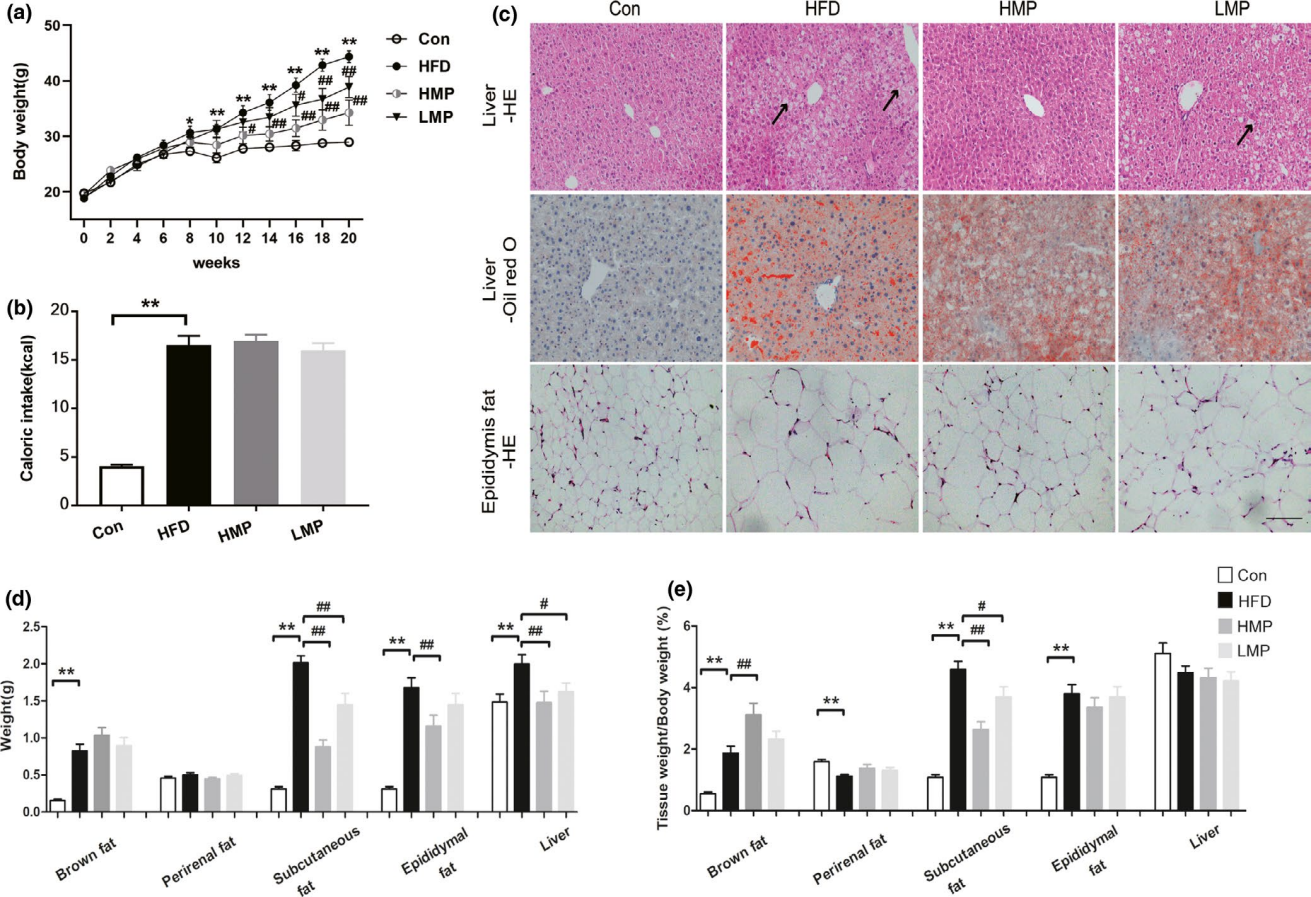
MP alleviates body weight and adiposity in HFD‐fed mice. (a) Body weight of the chow‐ and HFD‐fed mice either untreated or treated daily with MP for 20 weeks. (b) Caloric intake of mice. (c) Representative pictures of H&E‐ and oil red O‐stained liver, and epididymis adipose tissue stained by H&E (scale bar, 200 μm). (d) Tissue weight and (e) the ratio of organ/tissue weight to body weight of mice in the three groups (*n* = 8–10, *p < .05, **p < .01, versus Con; #*p* < .05, ##*p* < .01, versus HFD)

The comparison of the body weight and 24 h caloric intake of the three groups is represented in Figure 1a,b. The HFD‐fed mice presented similar body weight to mice in control group in the beginning of the experiment and significantly gained body weight from the 8th week (*p* < .05, versus Con). HMP significantly decreased body weight compared to the HFD group at the 12th week from 34.26 ± 1.25 g to 30.15 ± 1.50 g (*p* < .05, versus HFD; Figure 1a). This decreasing trend continued until the 20th week from 44.37 ± 1.07 g to 34.26 ± 2.29 g (*p* < .01, versus HFD). In the LMP group, the body weight was also gently decreased (*p* < .01, versus HFD). Compared with Con group, the HFD‐fed mice had significantly more caloric intake. MP supplementation did not affect caloric intake significantly (Figure 1b). Administration of MP markedly attenuated the HFD‐induced hepatic steatosis demonstrated by alleviating tissue cavitation and lipid droplet aggregation. The volume of epididymal adipocytes was also decreased by HMP treatment (Figure 1c). Consistently, HFD significantly increased weights of the organ/tissue, including brown fat, subcutaneous fat, epididymal fat, and liver. HMP administration reduced the mass of subcutaneous fat, epididymal fat, and liver (*p* < 01, versus HFD). By contrast, HMP mildly increased the mass of brown fat, which is responsible for the consumption of energy to heat and helping lose weight (Figure 1d). Supplementation of low dose of MP (LMP) also decreased subcutaneous fat and liver weight (*p* < .01, *p* < .05, versus HFD). The proportion of adipose tissue weight to body weight showed the same trend (Figure 1e).

#### 3.2.2 MP improves glucose tolerance, insulin resistance, and plasma lipid level

**FIGURE 2:**
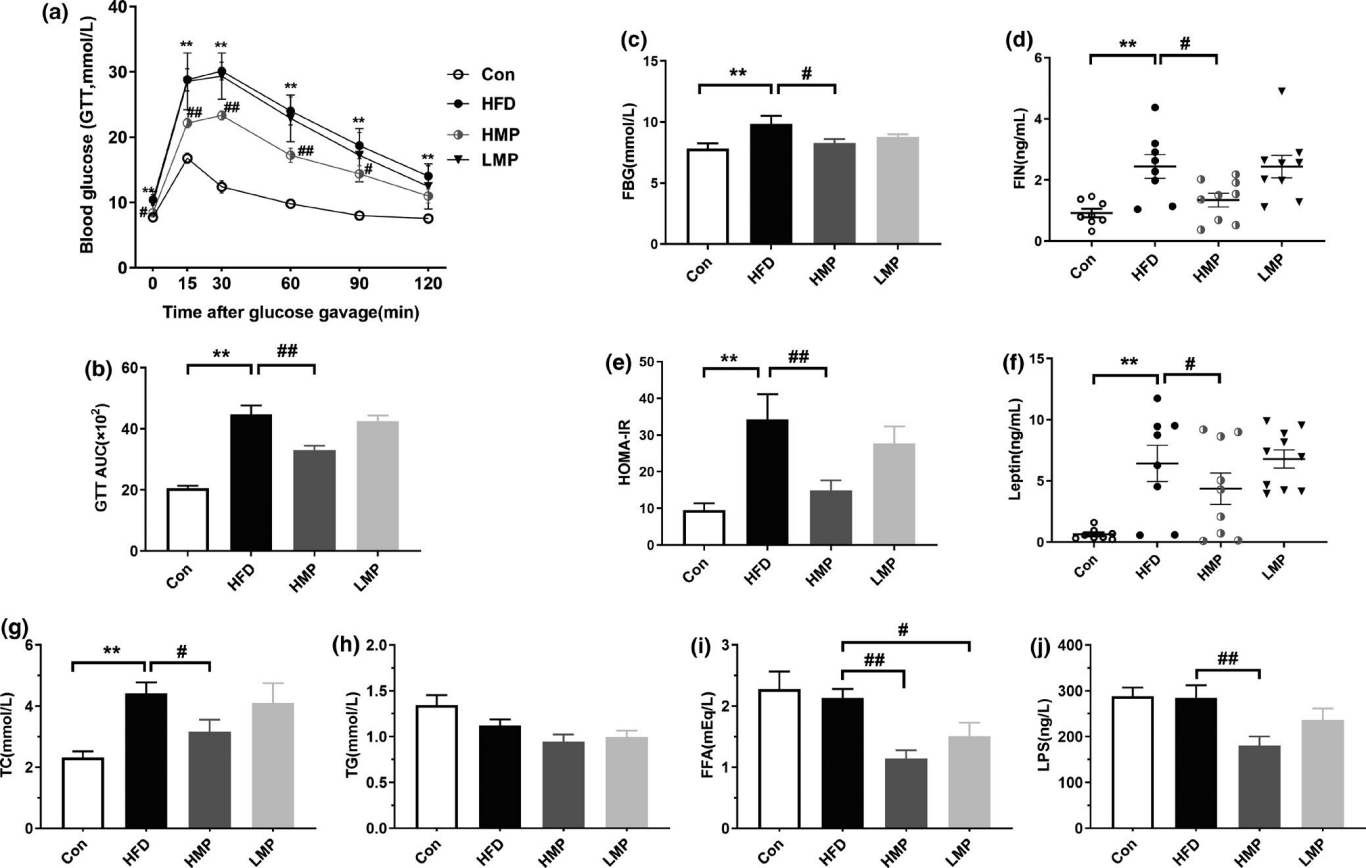
MP exhibits metabolic protection in HFD‐fed mice. (a) Effect of MP on glucose tolerance measured by glucose tolerance test (GTT) and (b) the area under the curve (AUC) of GTT (GTT AUC). (c) Fasting blood glucose (FBG), (d) fasting insulin (FIN), (e) homeostasis model assessment of insulin resistance (HOMA‐IR), plasma (f) leptin, (g) total cholesterol (TC), (h) triglyceride (TG), and (i) free fatty acid (FFA), and (j) lipopolysaccharide (LPS) levels in HFD‐induced obesity mice (*n* = 8–10, *p < .05, **p < .01, versus Con; ^#^ *p* < .05, ^##^ *p* < .01, versus HFD)

To evaluate how the body manages postprandial blood glucose by MP treatment, the GTT was performed (Figure 2a,b). Compared with Con group, HFD group mice exhibited a significant rise in blood glucose levels throughout 2 h post‐GTT and a maximum glucose concentration at 30 min. The decrease was present in HMP‐treated group, from 30.11 ± 1.35 mmol L^−1^ to 23.32 ± 0.68 mmol L^−1^ at 30 min (*p* < .01, versus HFD). Also, GTT AUC was higher in HFD mice than that of Con (*p* < .01) at the 20th week. HMP intervention decreased the GTT AUC from 44.67 ± 2.98 to 33.02 ± 1.44 (×10^2^) (*p* < .01, versus HFD). The enhancing effects of HFD feeding on FBG and FIN levels were observed (*p* < .01, versus Con), and insulin resistance was once more assessed. The results indicated that HMP markedly reduced FBG and FIN levels (*p* < .05, versus HFD; Figure 2c,d). HOMA‐IR was increased in the HFD group, indicating a lower insulin sensitivity compared with the Con group (*p* < .01, versus Con), while the index was reduced back to levels similar to the chow‐fed mice in the high dose of MP group from 34.24 ± 6.90 to 14.88 ± 2.76 (*p* < .01, versus HFD; Figure 2e), suggesting that HMP can alleviate IR and enhance insulin sensitivity in HFD mice.

Meanwhile, other related metabolic disorders, specifically including the leptin and total TC in the plasma, were markedly upregulated in HFD‐fed mice (*p* < .01, versus Con). HMP has been shown for the first time to markedly reduce the leptin level from 6.43 ± 1.49 ng ml^−1^ to 4.36 ± 1.28 ng ml^−1^ (*p* < .05, versus HFD; Figure 2f). Just as widely reported, HMP treatment dramatically alleviated TC in HFD mice from 4.40 ± 0.36 mmol L^−1^ to 3.15 ± 0.39 mmol L^−1^ (*p* < .05, versus HFD; Figure 2g; Zhang et al., 2014, 2018). Supplementation of low dose of MP did not statistically affect the value of GTT, FBG, FIN, HOMA‐IR, leptin, and TC. There was no significant increase in TG, FFA, and LPS observed in HFD‐induced mice in this study (*p* > .05, versus Con; Figure 2h–j). Surprisingly, FFA and LPS were largely reduced in HMP‐treated mice (*p* < .01, versus HFD). The same trend was also detected in the LMP‐treated mice.

### 3.3 MP ameliorates the pathological changes to colon tissue in HFD‐fed mice

**FIGURE 3:**
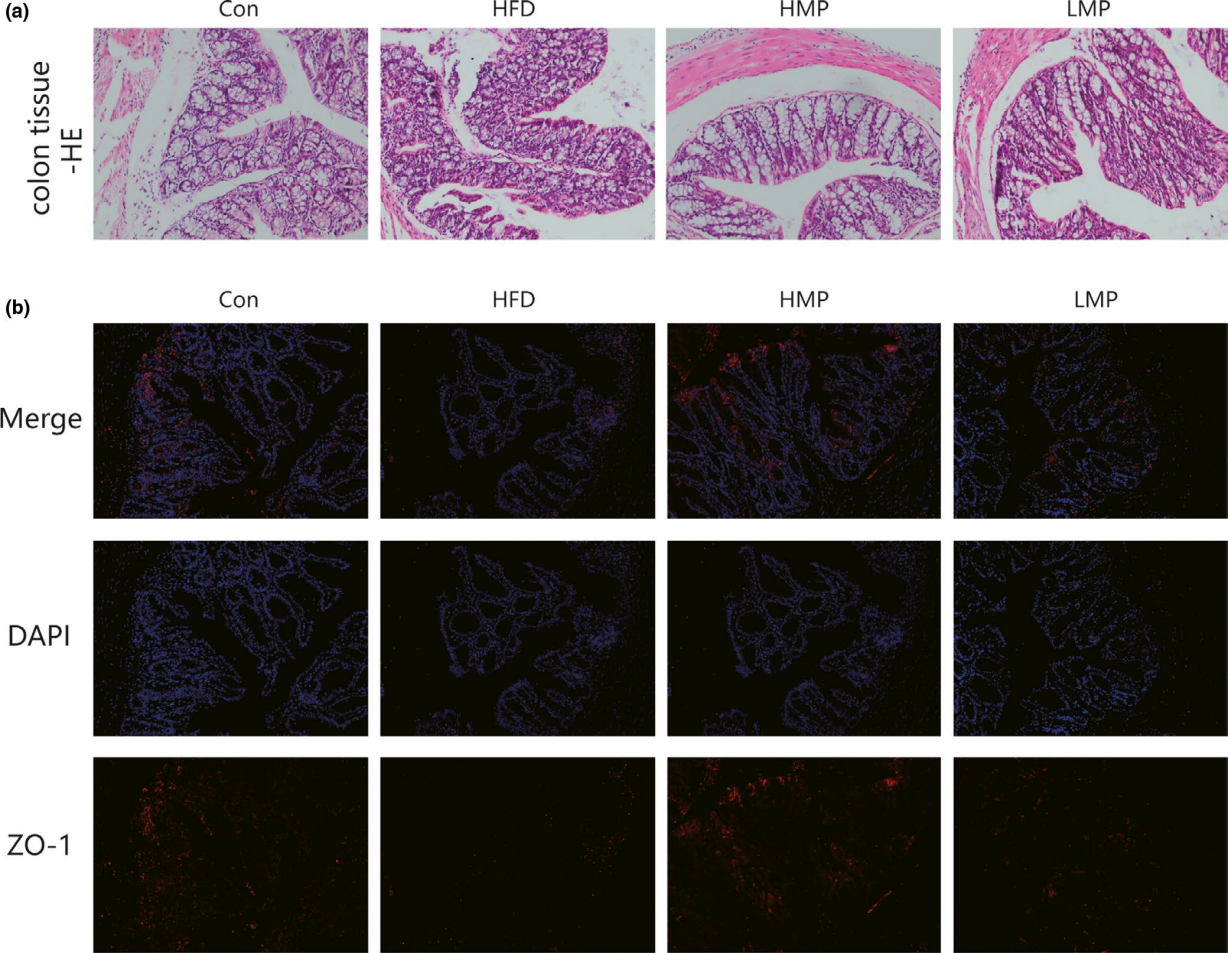
(a) Histopathological examination of colon tissue by H&E staining. (b) The immunofluorescence method was used to detect the protein expression of ZO‐1 in colon tissue. (×200 magnification)

The histopathological changes in each group are presented in Figure 3a. Compared with the Con group, the edema and connective tissue hyperplasia were observed in the submucosa of the tissue in the HFD group, with the interstitial space significantly enlarged, accompanied by a small quantity of focal infiltration of inflammatory cells. After treatment with high dose of MP (HMP), the phenomena described above were improved, exhibiting relative intact colonic architecture with less inflammatory cell infiltration. Immunofluorescence results demonstrated that ZO‐1 was principally expressed in the epithelial layer of the intestinal mucosa. Following treatment with HMP resulted in significantly higher fluorescence intensity of ZO‐1 protein. There was no obvious pathological change (versus HFD) in all tested colon tissue in the LMP group. The images obtained using fluorescence microscopy can be seen in Figure 3b.

### 3.4 MP enhances gut microbiota diversity and reduced the ratio of Firmicutes to Bacteroidetes (F/B) of HFD‐induced mice

**FIGURE 4:**
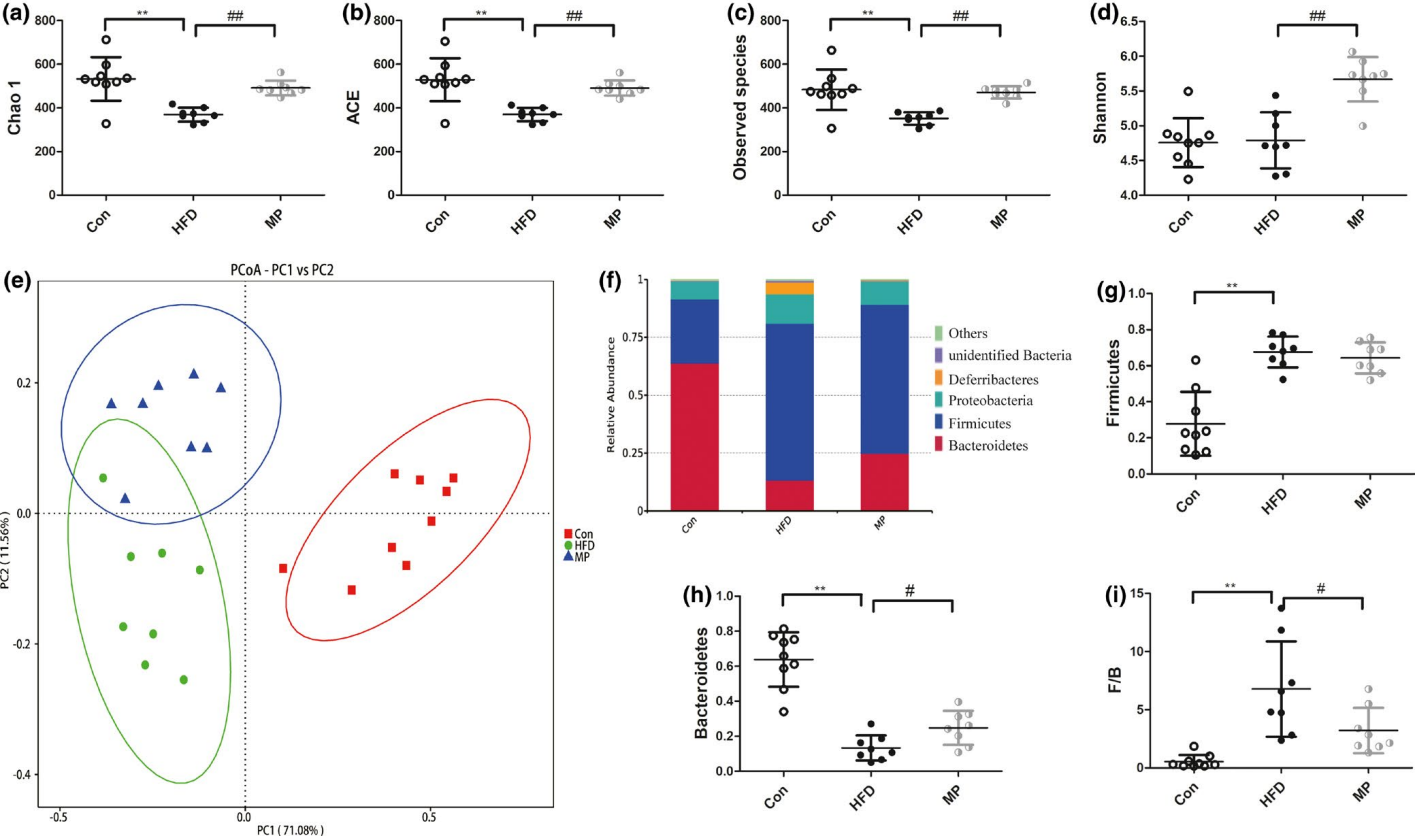
Alpha diversity analysis of different groups including (a) Chao1; (b) ACE; (c) observed species; and (d) Shannon index. (e) Beta diversity of weighted UniFrac‐based PCoA of different groups. The relative abundance of bacterial communities at (f) total phylum levels and (g) Firmicutes, (h) Bacteroidetes, and (i) F/B ratio (*n* = 8–10, *p < .05, **p < .01, versus Con; ^#^ *p* < .05, ^##^ *p* < .01, versus HFD)

Alpha diversity analysis, including chao1, ACE, observed species, and Shannon, was used to describe the complexity and species richness of bacteria in different samples (Figure 4a–d). HFD led to a decreased gut microbiota diversity. Compared with the Con group, the chao1, ACE, and observed species index were significantly lower in the HFD group (*p* < .01, versus Con). But no difference was observed in the Shannon index between Con and HFD group. By treatment with high dose of MP, the chao1 index was significantly increased from 368.8 ± 11.29 to 491.6 ± 12.05 (*p* < .01, versus HFD). The same enhancing trend was also detected after MP treatment in ACE (from 369.8 ± 10.86 to 491.3 ± 12.38), observed species (from 351.6 ± 10.02 to 471.1 ± 9.92), and Shannon index (from 4.8 ± 0.14 to 5.7 ± 0.11). Weighted UniFrac‐based principal coordinates analysis (PCoA) indicated the obvious difference between the HFD and Con groups after the 20‐week high fat diet, and the MP samples located between the HFD and Con samples (Figure 4e). At the phylum level, Bacteroidetes, Firmicutes, Proteobacteria, Deferribacteres, and unidentified Bacteria are the most abundant bacteria in mice gut microbiota (Figure 4f). Compared with Con group, HFD‐fed mice possessed more Firmicutes (*p* < .01), but less Bacteroidetes (*p* < .01). After MP intervention, the gut microbiota composition was altered toward that of the Con group, as can be seen in the significantly decreased ratio of F/B (*p* < .01, versus HFD; Figure 4g–i).

### 3.5 Correlations between the key microbial phylotypes and MP‐related parameters

**FIGURE 5:**
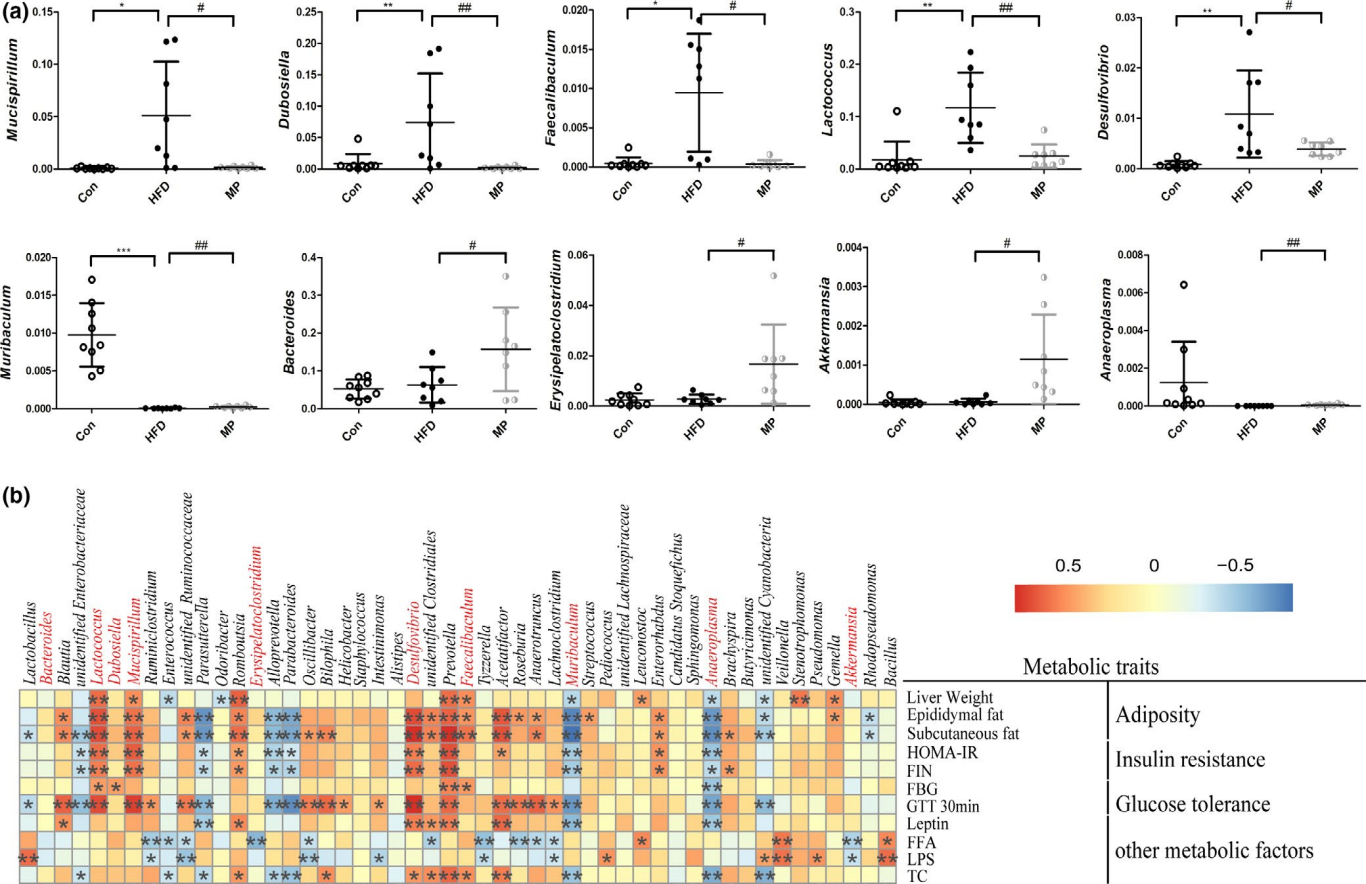
(a) Statistical analysis of genus whose abundance is significantly different among different groups. The *y*‐axis shows the relative abundance of corresponding genus. (b) Spearman's correlation analysis between 50 identified bacterial species and metabolic traits (*n* = 8–10, *p < .05, **p < .01, versus Con; ^#^ *p* < .05, ^##^ *p* < .01, versus HFD)

At the genus level, compared with Con group, HFD group featured more *Mucispirillum, Dubosiella, Faecalibacullum, Lactococcus,* and *Desulfovibrio* (*p* < .05), but less *Muribaculum* (*p* < .001). When MP was supplemented to the HFD‐fed mice, the abundance of *Mucispirillum, Dubosiella, Faecalibacullum, Lactococcus,* and *Desulfovibrio* was decreased (*p* < .05) and that of *Muribaculum, Bacteroides, Erysipelatoclostridium*, *Akkermansia,* and *Anaeroplasma* increased (*p* < .05; Figure 5a). In order to find the relationship of the key bacteria and metabolic parameters, spearman correlation analysis was applied (Figure 5b). Upon the significantly reduced genera by MP, *Mucispirillum*, *Lactococcus*, *Faecalibaculum*, and *Desulfovibrio* were positively related with IR and adiposity indexes. The opposite correlations were observed for *Muribaculum* and *Anaeroplasma*. Specifically, *Desulfovibrio* correlated positively with most of the parameters of metabolic disorders in this study.

### 3.6 MP improves lipid profile of HFD‐fed mice by lipidomics analysis

**FIGURE 6:**
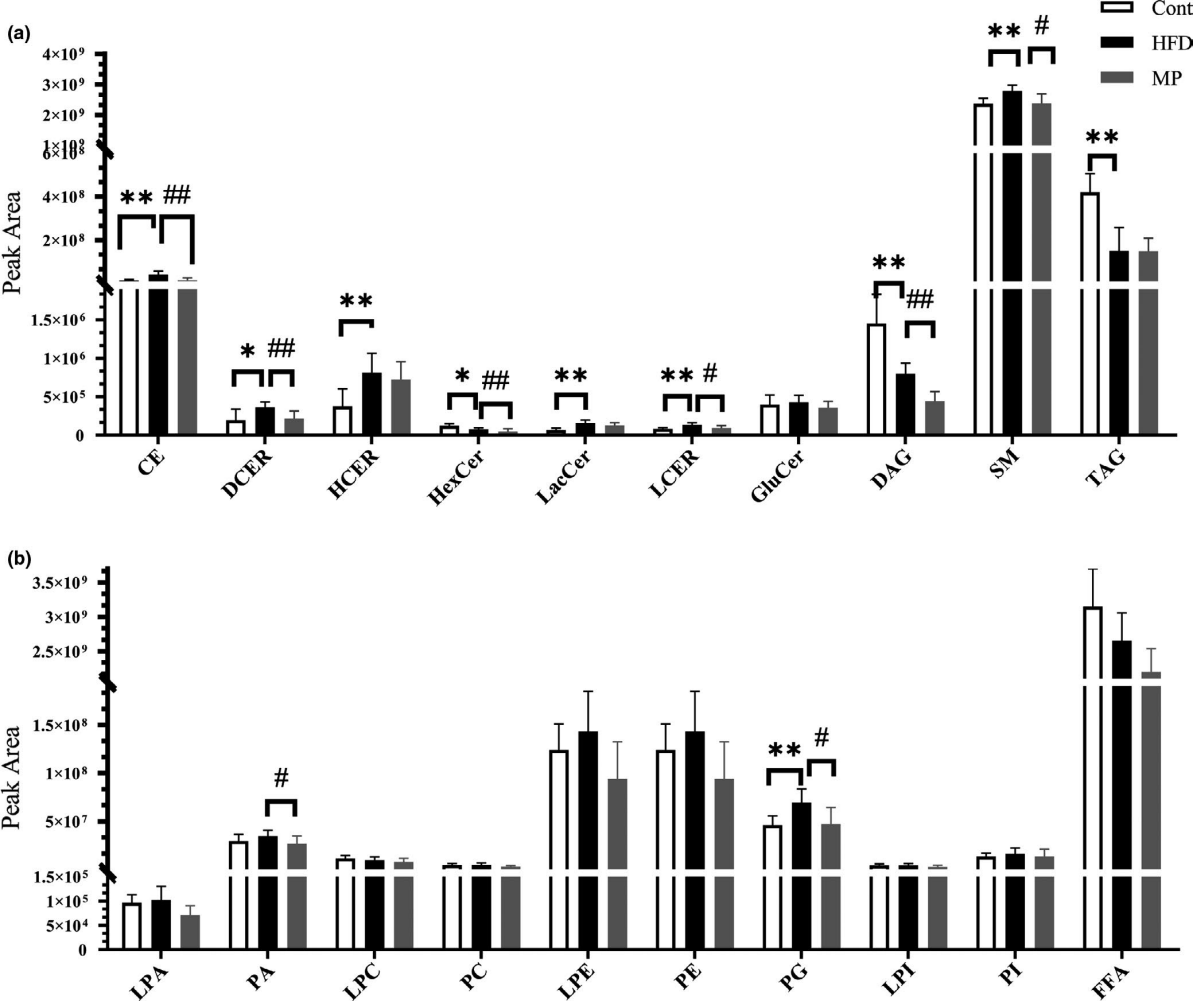
Histogram of various lipids. (a) The positive model. (b) The negative model (*n* = 8–10, *p < .05, **p < .01, versus Con; ^#^ *p* < .05, ^##^ *p* < .01, versus HFD)

For the lipids analysis, 20 types of lipids, including cholesteryl ester (CE), dihydroceramides (DCER), hexosylceramides (HCER), hexosylceramides (HexCer), lactosylceramides (LacCer), lactosylceramides (LCER), glucosylceramides (GluCer), diacylglycerol (DAG), sphingomyelin (SM), triacylglycerol (TAG), lyso‐PA (LPA), phosphatidic acid (PA), lyso‐PC (LPC), phosphatidylcholine (PC), lyso‐PE (LPE), phosphatidylethanolamine (PE), phosphatidylglycerols (PG), lyso‐PI (LPI), phosphatidylinositol (PI), and free fatty acid (FFA), were detected (Figure 6). HFD mice showed a significant increase in CE, DECR, HCER, LacCer, LCER, SM, and PG levels compared to the control group (*p* < .05), and CE, DCER, LCER, SM, and PG levels were reversed significantly by high dose of MP (*p* < .05). Besides, the levels of HCER, LacCer also showed decreased tendency in MP group. HFD mice showed a significant decrease in HexCer, DAG, and TAG compared with the control group (*p* < .05), which was consistent with that of plasma FFA detected by ELISA Kits.

**FIGURE 7:**
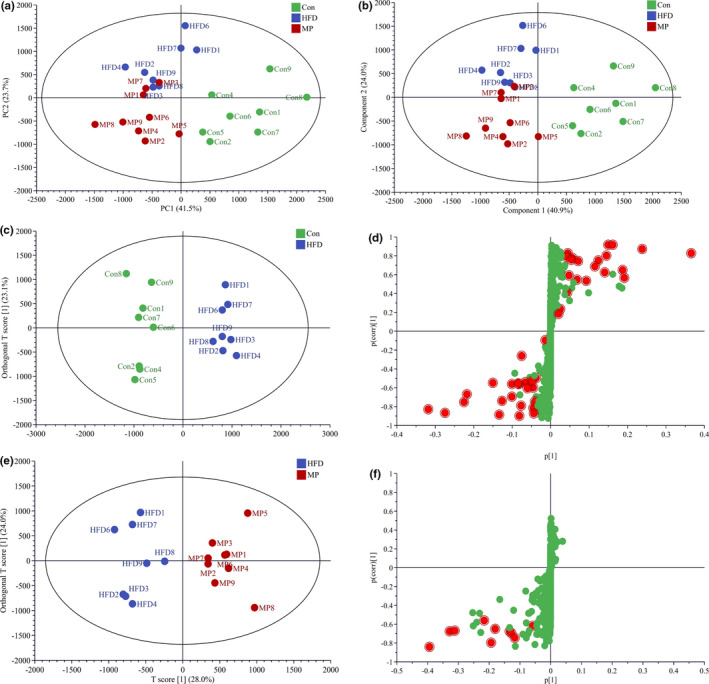
(a) PCA score plots. (b) PLS‐DA score plot. (c) OPLC‐DA score plot between control and HFD. (d) OPLS‐DA loading S plots comparing features between Con and HFD group with the lipids selected for biomarkers colored in red. (e) OPLS‐DA score plot between HFD and MP groups. (f) OPLS‐DA loading S plots comparing features between HFD and MP group with the lipids selected for biomarkers colored in red of the positive and negative models (*n* = 8–10)

PCA was performed to observe the clustering features among different groups. PC1 and PC2 accounts for 41.5% and 23.7% of the variance. It was observed that the HFD group clearly distinguished from the Con group, while the MP group separated away from the HFD group (Figure 7a). Likewise, the PLS‐DA model showed a clear separation among the Con, HFD, and MP groups, suggesting that lipid metabolic perturbations were induced by HFD and reversed by MP (Figure 7b). *R* ^2^ (describing the goodness of fit) for the model and *Q* ^2^ (estimating the predictive power after cross validation) for the prediction in the positive model (*R* ^2^ = .718, *Q* ^2^ = 0.685) indicated that the PLS‐DA model was reliable. The majority of samples in MP‐treated group were more close to the Con group, indicating that the consumption of MP improved the disordered metabolisms toward the normal status. The OPLS‐DA model and statistical analysis revealed a total of 42 variables between Con and HFD group (met variable importance factor VIP > 1 and *p* < .05 are identified as potential biomarkers; Figure 7c,d). These variables mainly belonged to the class of CE, FFA, PE, LPE, PG, PI, SM, and TAG, including one CE and PI, ten fatty FFA, two LPEs, six PEs, two PG, seven SM, and thirteen TAG. Notably, the large effect size was observed for FFA 16:1 (VIP = 9.56 and *p* = 1.04 × 10^–4^) and SM 16:0 (VIP = 8.21 and *p* = 1.94 × 10^–4^). After MP treatment in HFD mice, the level of CE (20:4), SM (18:0, 18:1, 20:0, 22:0, 22:1), LPE (18:2), PE (18:0/18:2), PG (18:2/16:1), and FFA (18:0) were remarkably downregulated (Figure 7e,f).

### 3.7 Correlations among the key microbial phylotypes, lipids, and MP‐related parameters

Pearson correlation analysis showed that the close association between the metabolic parameters and the lipids changed significantly by MP (Figure S2). Thus, these results indicated that lipids including CE, SM, LPE, PE, PG, and FFA might be potential biomarkers responsible for alleviating metabolic disorders of MP in HFD mice. Moreover, through Spearman correlation analysis between identified bacterial species and specific lipid (Figure S3), the genera upregulated by MP treatment including *Muribaculum*, *Akkermansia*, and *Anaeroplasma* were negatively correlated with lipid indexes, and the genera downregulated by MP including *Lactococcus*, *Mucispirillum*, *Desulfovibrio*, and *Faecalibaculum* showed positive correlation, indicating that MP might affect lipid metabolism via regulating gut microbiota.

## 4 DISCUSSION

Mulberry leaf is commercially sold and easily available as food and pharmaceuticals. With abundant nutritional constituents, mulberry leaf is a microecological modulator and has the potential of application in prevention and amelioration of obesity and other health problems. In this study, over 20 weeks of treatment, MP exhibits robust metabolic protective effect. We did not observe obvious side effects of MP in HFD‐fed mice. These data indicated that the 100 mg kg^−1^ day^−1^ MP (HMP) might be a promising therapeutic agent for metabolic disorders in terms of their efficacy and safety. We found that MP administration did not change calorie intakes. However, it prevented excess body fat accumulation, which contributed to adiposity control. Similarly, mulberry leaf significantly reduced body weight gain and fat mass of HFD mice (Li et al., 2011; Sheng et al., 2019; Xu et al., 2017; Zhang et al., 2018), indicating that the beneficial antiobesity effects of mulberry leaf may be associated with their functional polysaccharides. Besides, some compounds from mulberry leaf such as flavonols, quercetin, and rutin were reported to enhance energy metabolism (Chen et al., 2016; Kim et al., 2020; Stewart et al., 2008).

Obesity and related chronic inflammation states are often accompanied with increasing leptin concentrations and consequently lead to leptin resistance further fueling metabolic disorders (Ryan et al., 2020). These findings suggested that high dose of MP supplement might alleviate leptin resistance caused by an HFD. Most notably, the cause of IR in obesity and type 2 diabetes mellitus involves the complex interplay of multiple metabolic pathways (Yang et al., 2018), which is closely related with excessive specific lipids (including FFA) accumulation resulting in damage of insulin synthesis, secretion, and signal transduction (Liu et al., 2017). The lipidomics, emerged as a powerful tool, focus on analyzing the enormous complex lipid metabolites, and could assist to clarify specific lipid classes of MP treatment. Furthermore, gut microbiota plays a vital role in regulating lipid and glucose metabolism. LPS, mostly, gut‐derived LPS, promote white adipose tissue inflammation and contribute to impaired glucose metabolism (Caesar et al., 2012). Diet rich in fats is related to elevated systemic LPS level. Unfortunately, it did not work in our experiment, probably due to different fat content supplementing. MP treatment significantly mitigated metabolic endotoxemia, which is probably in association with gut microbiota regulation and gut barrier improvement. By the consumption of HFD, the gut barrier can become compromised, increasing intestinal permeability, especially to molecules of higher molecular weight, such as LPS, which contributes to the development of insulin resistance (Moreira et al., 2012).

MP treatment substantially shaped the microbial community in mouse gut. After MP intervention, the ratio of F/B was significantly decreased in the gut, which was in accordance with previous studies of mulberry leaf‐treating HFD‐induced obese mice (Sheng et al., 2019), and mulberry leaf component 1‐Deoxynojirimycin (DNJ)‐treating HFD‐induced nonalcoholic steatohepatitis (Hu et al., 2019). Some studies have described increases in the proportion of F/B in the obese phenotype compared with normal weight individuals, and a reduction in the proportion of the phylum Bacteroidetes was always associated with IR, obesity, and hyperglycemia (Chen & Devaraj, 2018). Given the first evidence of MP improving microbial diversity disturbed by HFD by this study, MP should be a therapeutic potential food via altering gut microbiota. Also, clinical studies are warranted to explore the beneficial effects on gut microbiota of MP.

Previous research has reported that HFD significantly increased the relative abundance of *Desulfovibrio* and elevated plasma LPS levels by inducing intestinal barrier dysfunction (Jakobsson et al., 2015). Accordingly, MP mice had significantly decreased circulating LPS levels when compared with the HFD mice, while there was no significant correlation between LPS and *Desulfovibrio* observed in this study (Chen et al., 2019). Increasing abundance of *Desulfovibrio* played an important role in high TC level, which can be reversed by lowering cholesterol treatment (Zhang et al., 2021). *Lactococcus* may be opportunistic pathogens in mice and that they would not cause physical damage in diabetic mice after MP treatment. We observed that the MP interventions significantly reduced the HFD‐accumulated species of *Faecalibaculum*, which is different from those reported previously (Ke et al., 2019). In addition, the reduction level of genera *Anaeroplasma* and *Muribaculum* was observed in diabetic mice compared with healthy mice (Yuan et al., 2020), and MP might reverse the gut microbiota dysbiosis by increasing their abundance. As a biomarker of an improved metabolic profile, *Akkermansia* contributed to lowering FFA and LPS level, indicating that MP might be against metabolic endotoxemia, adipose tissue inflammation through *Akkermansia* enrichment. This study provides new sights of microbial biomarkers alteration with increasing abundance of *Muribaculum, Akkermansia*, and *Anaeroplasma* and decreasing *Mucispirillum, Faecalibacullum, Lactococcus*, and *Desulfovibrio* by MP, which is expected to develop as a promising gut modulator for the treatment of metabolic syndrome. Similarly, accumulation of CE, SM, LPE, and PE in plasma of HFD‐induced obese mice has been reported (Eisinger et al., 2014; Zhai et al., 2018). SM is one of the major phospholipids; higher SM levels in plasma, liver, skeletal muscle, adipose tissue, and cardiovascular tissues were caused by HFD, which was involved in obesity‐induced IR, endothelial dysfunction, and atherosclerosis (Choi & Snider, 2015).

## 5 CONCLUSION

To conclude, MP was isolated and characterized with a range 1203–838 kDa and 0.3–203 kDa molecular weight and eight monosaccharides composition. The in vivo study using the HFD‐induced obese mice confirmed the beneficial effects of MP in improving metabolic disturbance and insulin resistance, gut microbiota composition, and lipid metabolism. Our results provide a scientific basis for the rational use of MP in clinic and suggest the development of MP as a functional food to treat metabolic disorder.

## CONFLICT OF INTEREST

The authors declare that they have no conflict of interest.

## Supporting information

Fig S1

Fig S2

Fig S3

## Data Availability

The data that support the findings of this study are available from the corresponding author upon reasonable request.
