# Supplementary figures and images for: Mulberry (Morus alba L.) leaf polysaccharide ameliorates insulin resistance‐ and adipose deposition‐associated gut microbiota and lipid metabolites in high‐fat diet‐induced obese mice
Source: Food Sci Nutr. 2021 Dec 24;10(2):617–30. doi: 10.1002/fsn3.2689 (PMC8825736; doi:10.1002/fsn3.2689)

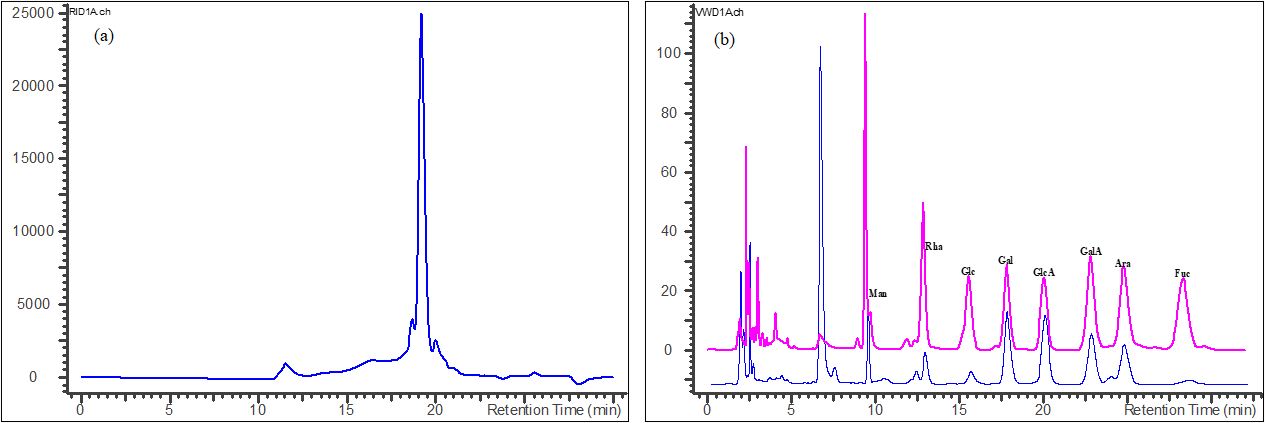

Supplement: Supplementary file 1 — Fig S1 [file FSN3-10-617-s003.tif]

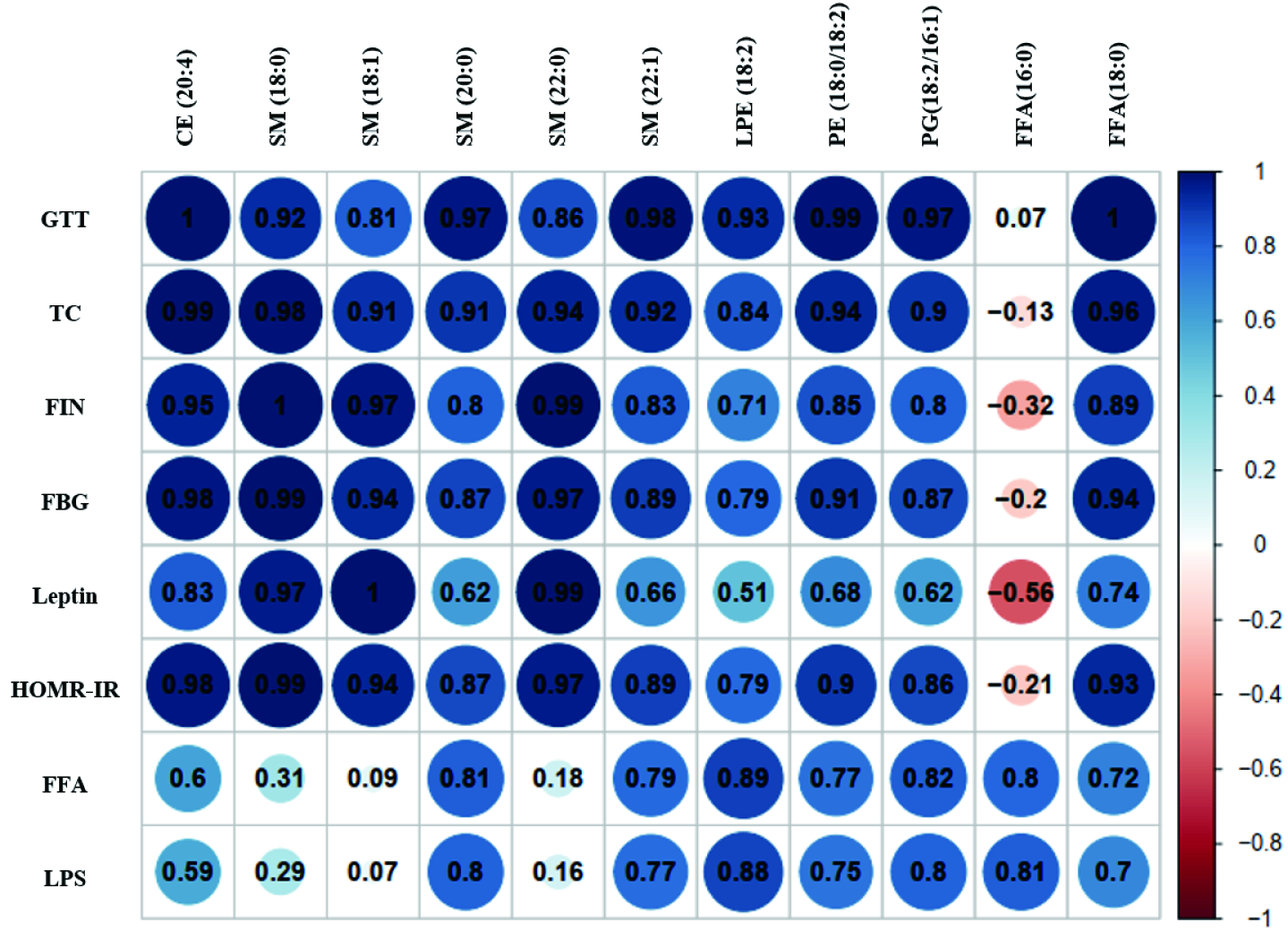

Supplement: Supplementary file 2 — Fig S2 [file FSN3-10-617-s002.tif]

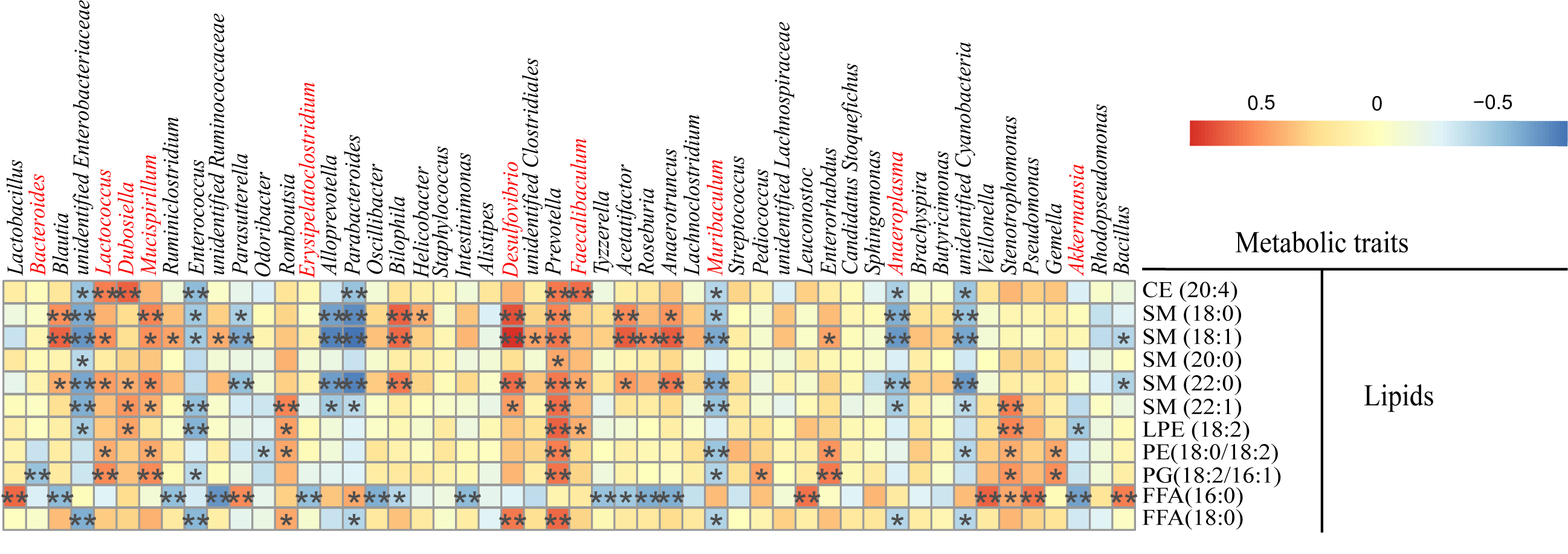

Supplement: Supplementary file 3 — Fig S3 [file FSN3-10-617-s001.tif]
